# Oxytocin receptor antagonism during early vocal learning reduces song preference and imitation in zebra finches

**DOI:** 10.1038/s41598-023-33340-7

**Published:** 2023-05-15

**Authors:** Natalie R. Pilgeram, Nicole M. Baran, Aditya Bhise, Matthew T. Davis, Erik N. K. Iverson, Emily Kim, Sumin Lee, Carlos A. Rodriguez-Saltos, Donna L. Maney

**Affiliations:** 1 Department of Psychology, Emory University, Atlanta, GA USA; 2 Present Address: Helen Wills Neuroscience Institute, University of California Berkeley, Berkeley, CA USA; 3 Present Address: Department of Integrative Biology, University of Texas at Austin, Austin, TX USA; 4 Present Address: Department of Biology, Hofstra University, Hempstead, NY USA

**Keywords:** Social behaviour, Animal behaviour

## Abstract

In species with vocal learning, acquiring species-typical vocalizations relies on early social orienting. In songbirds, for example, learning song requires dynamic social interactions with a “tutor” during an early sensitive period. Here, we hypothesized that the attentional and motivational processes that support song learning recruit the oxytocin system, which is well-understood to play a role in social orienting in other species. Juvenile male zebra finches naïve to song were each tutored by two unfamiliar adult males. Before exposure to one tutor, juveniles were injected subcutaneously with oxytocin receptor antagonist (OTA; ornithine vasotocin) and before exposure to the other, saline (control). Treatment with OTA reduced behaviors associated with approach and attention during tutoring sessions. Using a novel operant paradigm to measure preference while balancing exposure to the two tutor songs, we showed that the juveniles preferred to hear the song of the control tutor. Their adult songs more closely resembled the control tutor’s song, and the magnitude of this difference was predicted by early preference for control over OTA song. Overall, oxytocin antagonism during exposure to a tutor seemed to bias juveniles against that tutor and his song. Our results suggest that oxytocin receptors are important for socially-guided vocal learning.

## Introduction

Young animals face a multitude of choices about which objects in the world to pay attention to. During development, some stimuli acquire more relevance than others; these stimuli become “special”, or even rewarding^1,2^. Social stimuli are particularly relevant for many species and preferential orienting to social cues is critical for early social development. In human infants, for example, a “socio-motivational gate” is thought to bias attention towards a targeted social cue, such as a caregiver’s face or voice, resulting in the experience of social reward and facilitated development of socially acquired skills^3^. The acquisition of language in particular is thought to rely on social gating of incoming sensory information during dynamic, rewarding social interactions^1,4,5^.

In songbirds, learning to sing is similarly social. Decades of experimental work demonstrate that juveniles learn song most effectively from an adult “tutor” with which they can interact^6^. In zebra finches (*Taeniopygia guttata*), a species in which only the males sing, juveniles are most likely to choose their father as their tutor. This choice is clearly based on social, not genetic, factors, as young birds will copy the song of a foster father even when the biological father can be heard in the same room^7^. Even before they begin to sing, juvenile male zebra finches form strong preferences for the song of their adult male caregiver^8,9^. Young birds will key-press more to hear the song of their chosen tutor than to hear another familiar male, and the magnitude of this preference predicts how well that song will be learned^8^. These studies represent only a small fraction of a large literature suggesting that the mechanisms underlying song learning may share features in common with human vocal development—namely that song learning relies on social reward.

One of the key neuromodulators underlying social reward is oxytocin^10,11^. Although oxytocin is sometimes assumed to facilitate early orienting to a caregiver, including in humans, few studies have actually tested this hypothesis^12–14^. In juvenile mice, administration of an oxytocin antagonist early in life reduced motivation to return to caregivers after a period of separation^15^. Similarly, in newborn lambs, oxytocin antagonist reduced exploration of the mother’s body and impaired preference for her^16^. In visually naïve chicks (*Gallus gallus*), administration of the avian homolog of oxytocin increased motivation to approach hens^17^. Thus, the role of oxytocin in attraction to social cues may be conserved across species and developmental stages. Accordingly, we and others have hypothesized that, in songbirds, oxytocin receptors (OTR) mediate attraction and attention to tutor song, thus facilitating song learning^1,8,18–21^.

Birds have a clear homolog of OTR^22,23^; we will use the name “OTR” for that homolog, in keeping with established nomenclature^24,25^. OTR is expressed in many of the same brain regions in mammals and birds, including the lateral septum and auditory cortex^22,26^; in songbirds it is also expressed in song control areas^22,26^. We recently mapped OTR binding and mRNA in zebra finches throughout development, from day post-hatch (dph) five to adulthood, and found that this receptor is expressed in all of these regions throughout the entire period of song learning^18^. In the song system, OTR is expressed at higher levels in males than in females^18^. Together, these findings have formed the basis for our hypothesis that OTR facilitates song learning by directing attention to tutors, promoting the formation of social preferences, and guiding the development of selective encoding of tutor song^1,8,18,19^.

To show that OTR activity contributes to the formation of song preferences during development, and ultimately to song learning, we blocked OTR in juvenile, song-naive male zebra finches during early exposure to one of two song tutors. We used an antagonist known to effectively displace the endogenous ligands in brain tissue in this species^26^. Because zebra finches can produce accurate copies of songs after hearing as little as a few seconds per day over a few days^27,28^, we were able to limit exposure to live tutors precisely to times of pharmacological manipulation of OTR^29^. We hypothesized that pairing a tutor with OTR antagonism would reduce the reward value of that tutor’s song as well as the extent to which that song was ultimately learned. Thus, we predicted that the juveniles would prefer to hear the song of the control tutor (no OTR antagonism) over that of the tutor paired with OTR antagonism, and that they would more accurately copy the song of the control tutor.

## Results

### Oxytocin antagonism affected the pupils’ behavior during tutoring

**Figure 1:**
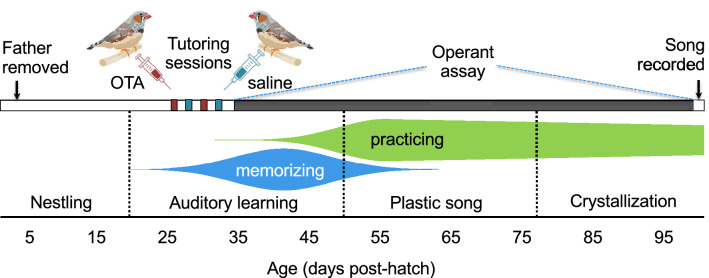
Experimental timeline and developmental trajectory of song learning in male zebra finches. Soon after fledging at ~ 18 days post-hatch (dph), male zebra finches enter an auditory phase of song learning during which they memorize adult song^32^ and start practicing singing^33^. Singing rates increase dramatically around age 50 dph. At approximately the same time, juveniles enter the “plastic song” phase^34,35^, during which some memorization may still occur but ends by 65 dph^32^. Song crystallization, during which the song takes its final form, begins by 77 dph and finishes by 90 dph^33,34^. In this study, male zebra finch “pupils” were reared in sound-attenuating chambers. Fathers were removed at 4 dph, such that pupils were thereafter isolated from male song until tutoring sessions commenced at 27 dph. During tutoring sessions, pupils were treated with either an oxytocin antagonist (OTA) during exposure to a tutor singing “OTA song” or treated with saline (control) during exposure to a tutor singing “control song” (see “Methods”). Treatments/tutors were alternated in counterbalanced order. At 35 dph, when they were nutritionally independent^33^, the pupils were transferred to an operant chamber equipped with keys that played tutor songs according to a reinforcement schedule that allowed us to measure preference while balancing exposure to the two songs (Fig. 3; see also ref. 8). Song preference, namely preference for control song over OTA song, was measured daily while the pupils remained in the operant chamber, until 99 dph. We recorded the songs of the pupils at 101–103 dph and compared them to the control and OTA songs. Illustrations of finches by DataBase Center for Life Science, shared under CC BY 4.0, https://creativecommons.org/licenses/by/4.0.

Song-naïve juvenile male “pupils” (n = 9) were tutored just prior to nutritional independence (27–33 dph) by two unfamiliar adult males in separate sessions (Fig. 1). Each pupil was assigned tutors with songs dissimilar to each other and to the father (see “Methods” for how the songs were compared). Before the start of each tutoring session, pupils were injected subcutaneously with either oxytocin antagonist (OTA, see “Methods”) or saline (control) in counterbalanced order. This antagonist, administered peripherally at the same dosage, was previously shown to reduce social approach^30^ and inhibit pair bond formation^31^ in adult zebra finches. In our study, each treatment was always associated with the same tutor; in other words each juvenile was exposed to an “OTA tutor” and a “control tutor” (note that it was the pupils, not the tutors, that were treated) in a within-subjects design. Here, we will use the terms “control song” and “OTA song” to refer to the songs heard by the pupil during each treatment condition.

**Figure 2:**
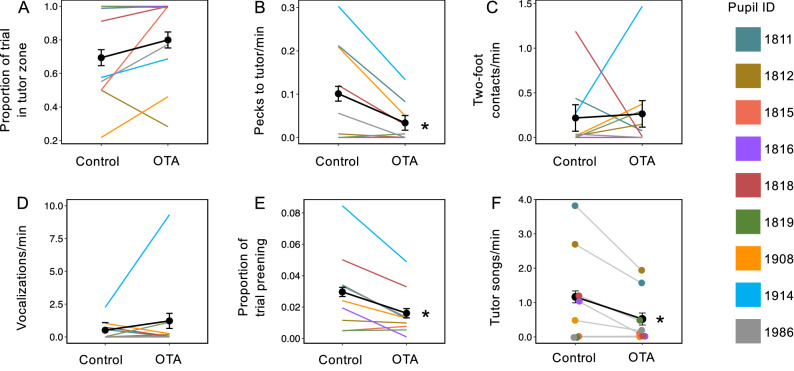
Effects of oxytocin antagonist (OTA) on behavior during tutoring. Approach-related pupil behaviors are shown in panels (**A**) and (**B**); attention-related pupil behaviors are in panels (**C**) through (**E**), and panel (**F**) shows a tutor behavior. There was no effect of OTA on the time spent close to the tutor (**A**). Regardless of treatment, pupils tended to spend most of their time in a “tutor zone” within 12 cm of the side of their cage closest to the tutor. OTA significantly decreased the number of pecks to tutor (**B**), or the number of times the pupil touched its beak to the wall of its own cage closest to the tutor (no pecks occurred on other walls in this study). There was no effect of OTA on bouts of flying, operationalized as two-foot contacts on cage walls (**C**), or on pupil vocalizations (**D**). OTA decreased the percentage of time pupils spent preening (**E**), which may be related to attention^37^. Tutors sang at significantly lower rates when presented with OTA-treated pupils than when presented with control-treated pupils (**F**); however, the number of live tutor songs did not predict the pupils’ preferences or learning (see “Results”). In (**A**–**E**) n = 9 pupils; in F, n = 8 tutors. Colors represent individual pupils as per the key on the right. All panels show mean scores (in black) for saline and OTA trials. To accurately depict the within-subjects variation in the error bars, the between-subjects variation was removed using the *summarySEwithin* function in R (v4.04) which implements the method described by Cousineau^38^ and the correction factor described by Morey^39^. The plotted standard error of the mean (SEM) is therefore equal between conditions for each score. *p < 0.05. See Supplementary Tables S1and S2 for statistics. Data are presented in Supplementary Table S4.

To test for effects of OTA on behavior during tutoring (Supplementary Table S1), we quantified behaviors previously shown to predict song learning in this species^36,37^. First, we scored approach to the tutor, operationalized in two ways. Time spent close to the tutor was defined as being within 12 cm of the wall facing the tutor’s cage (approximately 1/3 of the cage, designated as “the tutor zone”). Pupils tended to remain in the tutor zone regardless of treatment (Fig. 2A), so we were unable to detect an effect of OTA on proximity to the tutor. However, we did find that OTA significantly reduced the number of times the pupil approached the side of its cage closest to the tutor and touched its beak against that wall, which we scored as “pecks to tutor” (Fig. 2B; note that pecks never occurred on other walls in either condition). Thus, OTA did have an effect on observable interactions with the tutor.

We also scored behaviors that may reflect attention to the tutor. Chen et al.^36^ found that song learning was enhanced when pupils sat quietly, without flying or vocalizing, during tutoring. Similarly, Houx et al.^37^ reported an inverse relationship between learning and both activity and vocalizations of the pupil. In this study, we detected no effect of OTA on bouts of flying, operationalized as the frequency of two-footed contacts with cage walls (Fig. 2C), or on vocalizations by the pupil (Fig. 2D). We did, however, find an effect on preening, a behavior that occurs while birds are sitting quietly and that has been previously linked positively to the quality of song learning^37^. OTA significantly reduced the amount of time the pupils spent preening (Fig. 2E), which may indicate less attentive behavior in the OTA condition.

### Treating pupils with oxytocin antagonist affected tutor song rate

OTA treatment of the pupils affected song rate in the tutors; the number of songs sung per minute by tutors when presented with OTA-treated pupils was significantly lower than when presented with control-treated pupils. We detected this effect two ways, first within-pupil (Supplementary Table S2) and second, within-tutor (Fig. 2F; Supplementary Table S2). We addressed any imbalance in the number of songs heard from each tutor by playing recorded tutor songs during the tutoring session such that the number heard from each tutor was fairly equal (see “Methods”); nonetheless, the fact that tutors sang less frequently to OTA-treated pupils raised the possibility that effects of OTA on song preferences and learning could have been caused by hearing fewer *live* songs from the OTA tutors. We therefore checked for relationships between the number of live tutor songs and measures of preference and learning; we did not detect any such effects (see below). We also detected no relationships between the number of live songs heard by pupils and subsequent learning of that song (see below).

### Oxytocin antagonism decreased preference for tutor song

**Figure 3:**
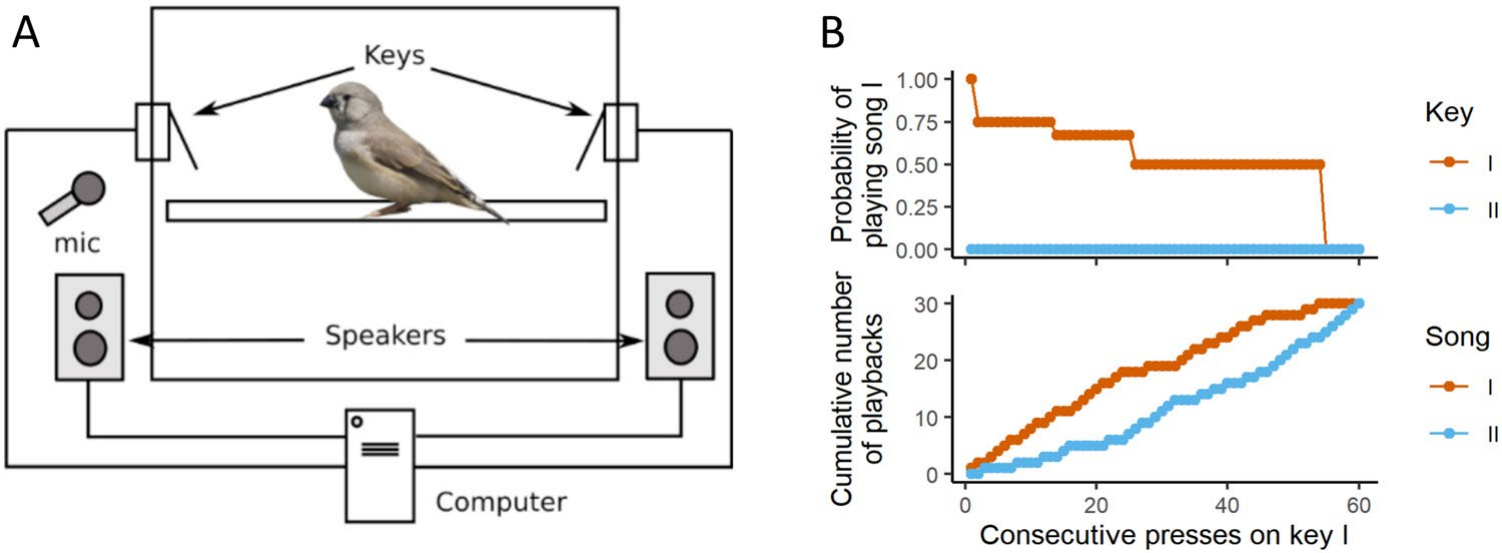
Key-pressing set-up and reinforcement schedule. (**A**) The operant chamber consisted of a 36 × 36 × 40 cm cage, inside which two keys were placed on opposite walls. One key was associated with playback of control song and the other with playback of OTA song. Outside the cage, one speaker assigned to each key played the songs. (**B**) demonstrates the reinforcement schedule. Keys I and II are associated with a higher probability of playing song I and song II, respectively^8^. Here we present an extreme example to illustrate how the schedule is able to balance exposure despite a strong preference. In this scenario, the bird prefers song I and presses key I only. The probability of playing song I by pressing that key is high at the beginning of the session, to help the bird form the association between that key and the song. As the bird keeps pressing key I, the probability decreases step-wise from 1 to 0.5, to prevent song II from lagging far behind in the playback count. This decrease, however, is not enough to balance exposure, and therefore, if the bird switches keys, key II plays only song II until the playback count of song II is balanced with song I. After enough presses on key I, song I eventually reaches a quota of 30 playbacks and ceases to be played. Afterwards, only song II is played, until that song also reaches the quota. Importantly, there is always a large difference between the keys with respect to the probability of hearing the preferred song. When the key associated with preferred song is playing that song only 50% of the time, the other key plays non-preferred song 100% of the time. The code used in this study, as well as an updated version, is available from Rodriguez-Saltos et al.^8^. Photo of finch “Australian zebra finch” by Lip Kee Yap, shared under CC BY-SA 2.0, https://creativecommons.org/licenses/by-sa/2.0. Background in the photo has been removed.

After two tutoring sessions with each of the two tutors (at 27, 29, 31 and 33 dph), pupils were moved (at 35 dph) into a cage containing two keys that triggered playbacks of tutor song when pressed (Fig. 3A). One of the keys was more likely to play control song, that is, the song of the tutor paired with saline treatment. The other key was more likely to play OTA song. Each day, the pupil could press the keys to trigger 30 playbacks of each song. Because of our novel reinforcement schedule (Fig. 3B; see “Methods”), exposure to the two songs was balanced each day. Each pupil heard 30 playbacks of control song and 30 of OTA song, after which the keys would trigger no further playbacks until lights-on the following day. After being transferred to the operant cages, the pupils engaged with the task and were exhausting the daily quota of 60 playbacks (30 of each song) within 5.5 ± 4.7 days (mean ± SD), a time frame similar to that reported by Rodriguez-Saltos et al.^8^ for juveniles that were reared until independence by their parents. Pupils remained in the operant chamber, completing the task each day, until 99 dph. One pupil was excluded from the study because of low levels of key-pressing; two others became ill (the illness was unrelated to the study) and were also excluded, leaving six pupils that completed the key-pressing portion of the study.

**Figure 4:**
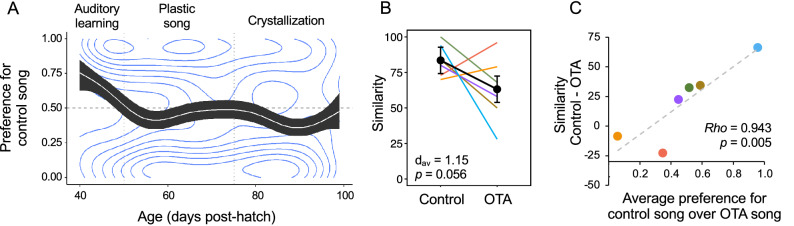
Effects of oxytocin receptor antagonist (OTA) during tutoring on song preference and learning. (**A**) Generalized additive model showing the trajectory of song preferences over the entire period of vocal development. The white line shows the average trajectory of the preference for the song of the tutor in the control condition, or ‘control song’. The black area indicates the 95% confidence interval (CI). Concentric blue lines represent the 2D density kernel estimation of the daily preference scores. Bandwidth was estimated via Normal Reference Distribution^8,41,42^. The horizontal dashed line at 0.5 indicates chance; values above the line indicate preference for control song and values below indicate preference for OTA song. Vertical dashed lines indicate boundaries between developmental phases (see Fig. 1). On average, preference for control song was significant during the auditory learning phase (CI does not include 0.5). Data are presented in Supplementary Tables S5 and S6. (**B**) Similarity between pupil song and tutor song was higher in the control condition than the OTA condition (maximum similarity is shown; for average similarity, see Supplementary Table S3). See caption of Fig. 2 for how the error bars were calculated. The effect size (Cohen’s *d*_av_) was large, at 1.15. See Supplementary Table S3 for statistics and Supplementary Table S7 for data. Colors represent individual pupils; see Fig. 2C The preference for control song over OTA song (averaged over 38–99 dph; plotted on the X-axis) significantly predicted the degree to which birds learned control song better than OTA song (Y-axis). Colors refer to individual pupils as per Figs. 2 and 4B. See Supplementary Table S8 for the values used to make the graph.

We estimated the strength of the preference for control song over OTA song daily by calculating the proportion of presses on the key associated with control song. Presses occurring after the quota of the preferred song was reached were excluded from this calculation. A generalized additive model showed that preference changed over time (*p* < 0.001; Fig. 4A). On average, control song was significantly preferred over OTA song (preference for control song > 0.5; 0.5 outside 95% confidence interval) between ages 40 and 47 dph, during the auditory phase of learning. At ~ 51 dph, average preference shifted toward OTA song, reaching an initial, significant peak at 55–59 dph, during the onset of plastic song. Preference for OTA song was significant again from 82 to 95 dph, during song crystallization. The pattern closely resembled that exhibited by normally-reared males, which on average prefer to hear their father’s song during auditory learning but then come to prefer a different song later, during plastic song and crystallization^8^. In the present study, many individual trajectories reflected this pattern in that they showed a peak in the proportion of presses for control song before 51 dph, followed by an increase in the proportion for OTA song after 51 dph (Supplementary Fig. S1). The strength of the preferences and the timing of these milestones varied from bird to bird as is common for developmental processes in other species, including humans^40^.

### Oxytocin antagonism inhibited song learning

For the six pupils that completed the key-pressing assay, we recorded their crystallized songs at 101–103 dph for comparison with both the control song and OTA song. Similarity scores, calculated with Sound Analysis Pro 2011^43^, were higher for control song than for OTA song. Although the p value for this effect was slightly above 0.05 (*p* = 0.056), the effect size was large *d*_av_ = 1.15; Fig. 4B; Supplementary Table S3). Thus, OTA treatment during exposure to a tutor appeared to impair learning of that tutor’s song.

### Song preference predicted song learning

We next tested whether the degree to which the pupils *preferred* control song over OTA song predicted the degree to which they *learned* control song more effectively than OTA song. We found a highly significant, positive correlation between the average preference for control song and the learning difference score, calculated as the similarity (see “Methods”) of the pupil’s song to control song minus its similarity to OTA song (*Rho* = 0.943; *p* = 0.005; Fig. 4C). Despite our low sample size, our power to detect this association was 0.92. This correlation between preference and learning remained significant even when limiting measures of preference to the period prior to song crystallization (38–70 dph) or to the auditory learning phase (38–50 dph; *Rho* and *p* were the same for both periods, *Rho* = 0.829; *p* = 0.042). The correlation persisted also within condition; preference for control song was positively correlated with similarity between the pupil’s song and control song (*Rho* = 0.829, *p* = 0.042 and negatively correlated with similarity to OTA song (*Rho* = -0.886, *p* = 0.019).

### Potential effect of live songs heard during tutoring

Because the pupils’ OTA status affected the song rate of the tutor, requiring more recorded songs to be played during the OTA condition on average, we next checked whether the reduced tutor song rate in the OTA condition (Fig. 2F) could explain the reduced preference for and learning of OTA song (Fig. 4A,B). First, we again tested for an effect of OTA on tutor song rate, this time limiting the sample to the birds for which we actually had data on preferences and learning (n = 6). In this case, there was no effect of the pupil’s OTA status on the number of live songs sung by the tutor (*X*^*2*^ = 1.189; *p* = 0.276). Further, there was a *negative* relationship between the proportion of live songs heard from the control tutor (vs. the OTA tutor) and the preference for control song, both over the entire trajectory of vocal development (*Rho* = − 0.600; *p* = 0.208) and early during development (38–50 dph; *Rho* = − 0.714; *p* = 0.111). Thus, we have no evidence that the preference for control song was caused by hearing more live song from control tutors. Similarly, the number of live songs heard from the control tutor did not significantly predict learning of control song (*Rho* = − 0.314; *p* = 0.544). Notably, the song of the pupil that heard only recorded songs from its OTA tutor was more similar to the OTA song than to the control song, and vice versa for the pupil that heard only recorded songs from its saline tutor. Thus, we have no evidence that the number of live songs heard, vs. recorded ones, affected learning in this study. We emphasize that all pupils self-tutored with 30 recordings of each song per day from 35dph to adulthood, so they were all exposed to the same number of recordings after live tutoring ended.

## Discussion

Here, we have shown that OTR antagonism during song tutoring both reduced preferences for that tutor’s song and inhibited learning of that song. This result is consistent with our hypothesis that OTR stimulation increases attraction to and attention to tutors during an early critical period, facilitating song learning. These findings add to a small but growing literature suggesting that OTR may promote early social bonds with caregivers, thus creating conditions conducive to enhanced social learning. Such a mechanism may be particularly relevant in the context of human language learning, which requires attention to a targeted social object such as a caregiver’s face or voice. In this way, vocal development may be ‘gated’ by dynamic social interactions^1,3–5^.

In addition to its effects on preferences and learning, OTA also measurably altered behavior during tutoring itself. We found that when treated with OTA, the pupils were less likely to engage in behaviors that may indicate interest in the tutor, namely approaching the tutor to peck on the cage wall. We also found that OTA-treated birds spent less time preening, a behavior done while sitting quietly. Both preening and quiet stillness were previously shown to predict the accuracy of song copying in zebra finches, perhaps because these behaviors facilitate listening^36,37^. OTR activation may promote self-grooming, particularly in social contexts. Other researchers have hypothesized that in mice, the oxytocin-dependent neural circuits that organize self-grooming are activated by social stimuli^44^. Preening as well as approach may be particularly relevant when these behaviors are contingent with singing by the tutor; Houx et al.^37^ reported that both these behaviors, when they occurred during playback of tutor song, correlated positively with the degree to which that tutor’s song was copied. Thus, although we did not analyze our current data with respect to contingencies, the timing of pupil behavior relative to tutor singing may be an important factor to consider in future studies.

The importance of tutor-pupil behavioral contingencies suggests that the tutoring context is best viewed as a dynamic environment in which both tutor and pupil play active roles. One of the most interesting results of the present study was that treatment of the *pupils* affected the behavior of *tutors*. When presented with OTA-treated pupils, tutors sang at lower rates than when presented with control-treated pupils. This finding suggests that tutors may have been attending specifically to oxytocin-dependent behaviors of their pupils. Other researchers have hypothesized that tutor song rate may be affected by pupil behavior^45^; however, there is little information on what those behaviors might be. Carouso-Peck et al.^46^ found that song learning was enhanced when tutor song followed pupil song. In our study, the tutoring sessions took place before the pupils began to practice singing, so we could not test for relationships between the song rates of tutors and pupils. In an exhaustive study of contingent tutor-pupil behaviors, Houx & ten Cate^45^ found no evidence that tutor song was predicted by any overt behavior from the pupil. Because of our small sample size and because we scored only a limited number of behaviors, we could not isolate a particular behavior in pupils that predicted tutor song rate. We also do not know the extent to which changes in tutor behavior, caused either by the pupils or by hearing their own recorded song through a speaker, might have affected song preferences and learning in the pupils^36,46^. Although we can say with confidence that preference and learning were not positively affected by the song rates of the tutors, we cannot rule out the possibility that OTA treatment of the pupils affected other tutor behaviors that, in turn, influenced the pupils’ tutor choices. Such influences are not easily teased apart in behavioral studies with live dyads. Nonetheless, our data implicate oxytocin receptors in the subtle dynamics of these dyadic interactions.

In another recent study using the same operant conditioning assay, we quantified preference for the male caregiver’s (father’s) song in juvenile male zebra finches^8^. In that study, pupils were naturally reared by their parents, both mother and father. At 35 dph, those pupils were transferred into an operant chamber with two keys, one of which was associated with the father’s song and the other with the song of a familiar “neighbor” that they had been able to hear but not see in their home cage during rearing. In that study, we found that the degree to which the pupils preferred the song they ultimately sang (which in that study was always the father’s song) strongly predicted the quality of learning of that song. That finding was replicated here; the degree to which each pupil preferred the control song over the OTA song significantly predicted the degree to which the pupil learned the control song better than the OTA song (Fig. 4C). Even though our sample size was small, this effect was dramatic. Together, these findings suggest that the ‘incentive salience’ of a signal early during development—in other words, its attractiveness—facilitates social learning of that signal.

Our current findings recapitulate the findings of Rodriguez-Saltos et al.^8^ in several other ways, including the shape of the developmental trajectory of song preference. In that study, the pupils demonstrated a significant preference for the father’s song during the auditory phase of learning. Then, at around 55 dph, their preferences shifted to the neighbor’s song, reaching statistical significance at 57–65 dph. Then, these males showed no preference for either song until crystallization, when there was another significant peak in preference for the neighbor’s song. Thus, the trajectory of preference for the father’s song, which is what all of the pupils ultimately sang in that study, was nearly identical to the trajectory of preference for control song in the current study. In other words, the pupils in the current study appeared to treat the control song as if it were the song of their father, and the OTA song as if it were the song of a familiar male with whom they had no social bond.

The timing of the shift in preference from the father’s song to the neighbor’s song, or in this study, from the control song to the OTA song, is important. In both studies, the preference for the song that was ultimately learned crossed the 0.5 mark, in other words shifted to the other song, precisely at the time that juveniles leave the auditory learning phase and begin singing plastic song (Fig. 1). This time, around 55 dph, is a time of major change in the life of a juvenile zebra finch, potentially corresponding to puberty^33,47^. The young male finch is unlikely to memorize a new song that he hears after this time, preferring to practice the song that he has already chosen to learn^6,32^. His song rate increases dramatically^34^ and the syllables in his song begin to resemble those in tutor song^35^. At the same time, he begins social exploration, spending more time away from the parents and instead interacting with unrelated finches^48^. Thus, we interpret the shift in preference as a sign of social exploration, or wanting to hear songs other than the one the pupil has himself chosen to learn.

Post-hatch day 55 is significant also because at this time, OTR expression in the brain undergoes interesting changes. In two previous studies^18,19^, we quantified the expression of OTR mRNA in regions relevant to sociality or song learning, such as the lateral septum, the auditory forebrain, HVC (a song control nucleus), and the dorsal arcopallium, which makes up part of the ‘shelf’ region around another song nucleus, the robust nucleus of the arcopallium. We found that in each of these regions, OTR mRNA expression declined precipitously around 55 dph. Expression then rebounded to previous levels by 65 or 75 dph. We hypothesize that this decrease, which was widespread in the brain yet limited to a relatively short time window, plays a role in the co-occurring shift in social preferences and behaviors.

This is not the first study to show effects of nonapeptides on song learning. In addition to OTR, songbirds also express receptors homologous to the vasopressin receptor V1aR in the brain^18,22,26^ and these receptors have been implicated in early sociality and song learning. Baran et al.^49,50^ treated zebra finch hatchlings with daily intracranial injections of Manning Compound, a V1aR antagonist, and tracked their behavior until maturity. Treated birds exhibited atypical social development and showed no clear preference to affiliate with their parents versus unfamiliar conspecifics. Treated males were also impaired in their song acquisition, and the degree of impairment was correlated with measures of atypical social behaviors^1^. Therefore, like OTR, V1aR may play a role in early attachment and song learning.

A limitation of this study was that we administered OTA subcutaneously rather than directly into the brain, so it could have acted peripherally to alter behavior and learning. In other species, including chickens, OTR can be found in a variety of peripheral tissues including the heart, vasculature, and adrenal glands^51,52^ and could affect heart rate and other processes related to stress^53^ The antagonist we used has been shown by others to affect social behavior in zebra finches when administered peripherally^30,31^; it will be necessary, however, to conduct central infusions, targeting particular receptor populations in the brain, to better understand the role of OTR in social reward during song learning. The binding pattern of the antagonist used in this study, ornithine vasotocin, resembles the distribution of OTR mRNA, not V1aR mRNA, regardless of age^18,22^. We therefore believe that if the antagonist used in this study is crossing into the brain, it is binding primarily to OTR rather than V1aR or another type of nonapeptide receptor. We cannot, however, make strong predictions about the relative displacement of the two endogenous ligands, oxytocin and vasopressin, by this antagonist. In birds, OTR is thought to serve as a common receptor for both nonapeptides^52^, which are predicted to bind equally well to this receptor^22^. Brain regions associated with sociality, such as the lateral septum and bed nucleus of the stria terminalis, contain dense immunoreactivity for both nonapeptides; regions associated with auditory processing and song learning, however, likely receive denser oxytocinergic than vasopressinergic input^21,54^.

Another potential limitation is that, because we were primarily interested in the effects of oxytocin antagonism on tutor choice, we included only males in this study. Others have shown that females also show preferences for their father’s song in choice tests. In adulthood, these preferences are just as strong as in males^55^ or even stronger^9^. OTR expression, namely in song control regions, is higher in males than females during the auditory learning phase^18^, suggesting that some OTR populations could be involved in song learning specifically. Future experiments should explore the possibility that social reward, particularly attraction to song, could rely on different OTR populations in female vs. male songbirds.

## Methods

### Animals

All procedures involving handling and experimentation with animals were approved by the Institutional Animal Care and Use Committee at Emory University under protocol 2003144-052615N. All methods were performed in accordance with the relevant regulations and guidelines (e.g., ARRIVE). Juveniles used in the study (n = 9), or “pupils”, were produced in our breeding colony at Emory University. All birds were housed under a 12-h-light/12-h dark cycle. Cages (36 × 36 × 40 cm) were kept individually in sound-attenuating chambers (interior dimensions 56 × 40 × 58 cm, Colbourn Instruments, H10- 24TA) to prevent exposure to adult songs. To discourage nestlings from developing a side bias that could affect our ability to measure song preference in the operant assay, all food cups and water bottles were matched by color and positioned symmetrically in the cage. The father of the nestlings was removed at four dph, counting from the hatch date of the oldest nestling, leaving the female to provide all parental care. After the father’s removal, therefore, hatchlings did not hear song until tutoring began (see Fig. 1). To ensure that all the pupils we selected were male and, therefore, could learn to sing, the sex of nestlings was determined using PCR analysis of DNA from blood samples^56^. Sex was later confirmed by plumage.

### Selection of tutors

We selected adult male tutors with high song rates. Song rate was determined by placing the cage of an adult male next to the cage of either an adult female or a juvenile male for one hour and noting the rate of singing. Even though each juvenile’s father was removed from the breeding cage by 4 dph, for each juvenile we nonetheless chose tutors with songs dissimilar to the father’s in order to limit potential bias towards learning one of the two tutor songs. This dissimilarity was assessed by visually inspecting spectrographs created in Audacity. We looked for shared “syllables”, or short, stereotyped sounds that are separated by a brief period of silence^33^. We matched each pupil to tutors that shared no more than two syllables with the pupil’s father (Range 0–2, M = 0.667, SD = 0.623). Moreover, for each pupil, the two tutors shared no more than two syllables with each other (Range, 0–2, M = 0.6, SD = 0.8). The shared syllables were primarily stereotyped, call-like sounds that typically vary little among individuals^33^.

### Song tutoring

Tutoring sessions took place at 27, 29, 31, and 33 dph, which corresponds to a period of high receptivity to tutoring^57^. Thirty minutes before each of four tutoring trials, pupils were treated with either sterile 0.9% saline (control) or 5 µg oxytocin receptor antagonist [d(CH2)51,Tyr(Me)2,Thr4,Orn8,des-Gly-NH29] -Vasotocin trifluoroacetate salt (Bachem) dissolved in saline. This dose of this antagonist was previously shown to inhibit pair-bond formation^31^ when administered peripherally in zebra finches. The same antagonist had similar effects on social approach, also in zebra finches, whether administered peripherally at this dose or centrally^30^. In this study, both OTA and saline were administered via a 0.05 mL subcutaneous injection into the shoulder area between the feather tracts. Trials were separated by ~ 48 h and the treatments, OTA or saline, were alternated. The order in which the pupils received each treatment was counterbalanced, such that some pupils received OTA first and some received saline first.

For each pupil, each of the two treatments was always associated with the same tutor; in other words, there was an “OTA tutor” and a “control tutor” for each pupil. Tutor pairs were each used for two pupils, with the identity of the control tutor and OTA tutor reversed. Pupils spent two tutoring sessions with each of their tutors, for a total of four sessions per pupil. Each tutoring session took place inside a walk-in sound-attenuating booth and lasted about an hour^29^. Immediately after receiving the subcutaneous injection of OTA or saline, a pupil was placed into a testing cage, brought into a booth, and given 30 min alone to acclimate to this environment before the tutoring session commenced. The tutor was given about 15 min to habituate in a separate, identical booth before being moved, in its cage, to the booth with the pupil. The tutor and pupil remained in separate cages during the tutoring sessions but could interact visually and vocally. We defined the start of the tutoring session as the moment when the experimenter left the room after placing the tutor in the booth with the pupil. Audio and video recordings were made of all tutoring sessions using a camera placed inside the booth.

We took steps to balance the number of times each pupil heard each tutor’s song during tutoring sessions, such that each pupil heard roughly the same number of songs from each of the two tutors. On the basis of our calculations of each tutor’s singing rate, which was made when selecting the tutors (see *Selection of tutors, above*), we estimated the approximate number of songs that we expected each pupil to hear during each of the four sessions. If this number was not reached by 45 min into the session, recordings of that tutor’s song were played from a speaker at 75 dB during the last ~ 15 min of the tutoring session. When the tutor sang at a rate higher than anticipated, tutoring sessions were cut a few minutes short in order to control the number of songs heard by the pupil. The average session duration was 58.6 min ± 5.0 min. Recordings were played in a large majority of sessions (86%). For ten of the sessions (six control and four OTA), the tutor did not sing and all songs heard by the pupil during that session were playbacks. As a result, there were three pupils that did not hear live songs from one of their tutors; in two cases, that tutor was the control tutor and in the other case it was the OTA tutor. After each session, the pupil and the tutor were brought back to their respective home cages.

### Behavioral observations

After all the trials were completed, the recordings were observed using Behavior Observation Research Interactive Software (BORIS, v.7.9.19). We focused on behaviors that others have previously found to be predictive of song learning, such as approaching the tutor^37^ and paying attention to the tutor^36^. To quantify approach to the tutor, we scored two behaviors. First, we quantified the percentage of total time during the session that the pupil spent close to the tutor, defined as within an area ≤ 12 cm from the wall of its own cage that faced the tutor’s cage. This area of the cage was designated as the “tutor zone.” Our second measure of approach was “pecks to tutor”, which was counted each time the pupil approached the wall of its own cage closest to the tutor and pecked at that wall.

Attention during tutoring, or more specifically, lack thereof, has been operationalized by others as the level of intense activity, such as flying, and number of vocalizations by the pupil, which both negatively predict learning^36^. To quantify flying, we scored a behavior that occurred frequently in the most active pupils: two-foot wall contacts, in other words when the pupil flew and landed with both of its feet on any side wall of the cage. Because the cages were 36 × 36 × 40 cm, there was little opportunity to remain airborne for extended periods of time or fly longer distances; thus, counting the number of times a bird alit on the sides of the cage served as a proxy for flying bouts. In addition, we counted all vocalizations made by the pupils. These vocalizations did not include song, as the tutoring sessions took place largely before juveniles begin producing even immature song^7^. As a final measure of attention, we also scored preening, a grooming behavior that we defined as when the pupil used its beak to clean its feathers or another part of its body. Preening during tutoring has been found to be positively associated with learning, possibly because the bird sits quietly while doing it^37^. In addition to the pupil’s behavior, we also scored singing by the tutor.

To test for effects of OTA treatment on behavior during tutoring, we used linear mixed models (LMMs) in R (v4.04). Separate tests were conducted for each measure. In all models, pupil ID was included as a random effect to accommodate the within-subjects design. For all variables, we used the following model: ~ Trial + Treatment + (1|Pupil ID), which allowed us to control for the effects of Trial (i.e. the first or second tutoring session for each treatment). To perform the LMM analyses, we used the *lmer* function in the lme4 package^58^. To test for a significant effect of Treatment on each variable of interest, we performed a likelihood ratio test using the *anova* function to compare the full model to a reduced model with Treatment removed. As a measure of effect size, we calculated Cohen’s d_av_ as described by Cumming^59^ and Lakens^60^ which takes into account the within-subjects design.

### Auditory stimuli

A unit of song contains two consecutive repeats of a highly stereotyped motif^5,33^. To create the playbacks used during tutoring sessions (see *Song Tutoring*, above) and in the preference assay (see *Operant assay of song preference*, below), two consecutive motifs from each tutor were isolated in audio files. Male zebra finches typically produce a series of call-like introductory notes immediately before beginning to sing; these introductory notes were excluded. The files were then cleaned using Audacity as described by Rodriguez-Saltos et al.^8^.

### Operant assay of song preference

Pupils were removed from their home cage at 35 dph, 48 h after the last tutoring session concluded, and housed singly in a cage inside a sound-attenuating chamber. By this age, juvenile zebra finches are able to feed themselves independently^33^. The operant set-up was as described by Rodriguez-Saltos et al.^8^. Two keys, accessible from a perch, were placed on opposing walls. (Fig. 3A). Each key was paired with one of two speakers, which were located inside the chamber on opposing sides. The speakers were both set to play song at 75 dB as measured at the center of the cage. A small mirror was affixed to the center of the cage’s back wall to provide social enrichment.

The operant assay ran on a probabilistic schedule of reinforcement controlled by the custom-written software program SingSparrow^8^. The program allowed the pupil to “self-tutor” by pressing a key to trigger a playback. When a playback was triggered, one song composed of two motifs played through the associated speaker. Juveniles have been shown to learn song effectively using this self-tutoring method, even with hearing as few as 15 songs each day^27^. Our operant schedule was designed to balance exposure to the two tutor songs, limiting each to 30 playbacks, while still allowing us to quantify preference for a particular song each day (Fig. 3B). This “self-balancing” schedule was designed as follows: both keys could play both songs, but each key was associated with a higher probability of playing one song or the other. As a key was pressed repeatedly, the probability that it would play its associated song decreased stepwise from 100 to 50%. Each key was, however, always more likely to play its associated song than was the other key, as long as the quota of 30 for that song had not been met. Additionally, whenever the pupil switched between keys, the first press following the switch always played that key’s associated song. After 30 playbacks had been triggered of one song, both keys played only the other song—the song in deficit—until all 60 songs were exhausted. At this point, the pupil could continue to press the keys, but no song would play.

On 36 dph, or the day after the pupil was isolated in an operant chamber, the keys were activated to begin the operant assay at lights-on. The assay ran through 99 dph, with the keys being reactivated every day at lights-on. The number of presses on each key was automatically logged between lights-on and lights-off each day, as well as which song played. To detect possible side biases, reversals were run as described by Rodriguez-Saltos et al.^8^.

### Analysis of song preference

The strength of a pupil’s preference for a particular song was determined by counting the number of presses per day on each key^8^. Data were excluded from any days during which a pupil’s total number of presses was fewer than five; these days happened early during training when the pupils were still learning the task. Daily counts of key presses were then filtered to include only those presses that occurred up to the point in the schedule when the quota of 30 playbacks for one song had been reached. After this point, the pupil no longer had a choice of which song to hear, making key presses less behaviorally meaningful. Key presses were removed also if they were triggered accidentally by the caretaker or by the bird while care was being provided.

After the data were cleaned as described above, we calculated daily “preference scores” for each pupil as a proportion of presses on one of the keys, e.g.: Key A presses/(Key A + Key B presses). Preference scores were calculated as the preference for the key associated with the song of the control tutor (“control song”; preference for OTA song is preference for control song subtracted from 1). To reconstruct the average trajectory of preference for control song, we fitted a generalized additive model^61^ to our data. The dependent variable was preference for the control song and the independent variable was age in days. Pupil identity was modeled as a random effect. To constrain predicted values to the interval 0–1, we modeled the dependent variable using a beta regression. The relationship between preference and age was modeled using a thin-plate regression spline^62^. The model was fitted using the library *mgcv*^63^ in R (R Core Team, 2019). *Mgcv* produced a 95% confidence interval of the trajectory by multiplying the standard error of the trajectory by two, subtracting this result from the trajectory to find the lower bound, and summing it to the trajectory for upper bound^63^. At any given age the pupils significantly preferred the control song over OTA song if the trajectory was above 0.5 and the confidence interval excluded that value.

### Quantification of song learning

Songs were recorded at 101–103 dph, the point at which pupils have completed their vocal development and produced a crystallized song that does not change much thereafter^33,34,64,65^. All songs used for this analysis were cleaned to remove introductory notes, etc. as described above. Song recordings were imported as .WAV files into Sound Analysis Pro 2011 (SAP). To calculate similarity between tutor and pupil songs, we segmented songs into syllables on the basis of an amplitude threshold^8,66^. Thresholds were adjusted for every song because each one was sung at a different distance from the microphone; thus, song-specific amplitude thresholds allowed for each song to be properly segmented. Similarity scores were calculated using SAP’s “Song Similarity” tool, which compares the acoustic features of one song’s syllables to another and quantifies similarity^66^. Similarity scores were calculated for each pupil-tutor pair using three to six song exemplars per pupil. Because we were primarily interested in the highest degree of match a pupil was capable of, rather than the pupil’s propensity to sing one song vs. the other, we used the maximum similarity score for each pupil-tutor pair in our analyses^67–69^. The average similarity scores are also presented and the results of those analyses are largely the same (see Supplementary Table S3).

To test the effect of OTA on song imitation, we compared similarity scores across condition for each exemplar using LMMs as described above (*Behavioral observations*) using the following model: ~ Treatment + (1|Pupil ID), where Pupil ID was included as a random effect. To test for relationships among factors such as song preferences, song learning, and the number of live tutor songs heard, we used Spearman correlation tests (SPSS v.28). Power analyses were done using G*power (v. 3.9.6).

## Supplementary Information

Supplementary Information 1.

Supplementary Information 2.

## Data Availability

The datasets supporting this article have been uploaded as part of the supplementary material. Code used for the operant assay was published by Rodriguez-Saltos et al.^8^.
