# Supplementary material for: Oxytocin receptor antagonism during early vocal learning reduces song preference and imitation in zebra finches
Source: Sci Rep. 2023 May 15;13:6627. doi: 10.1038/s41598-023-33340-7 (PMC10185528; doi:10.1038/s41598-023-33340-7)
Supplement: Supplementary file 1 — Supplementary Information 1. [file 41598_2023_33340_MOESM1_ESM.pdf]

**Oxytocin receptor antagonism during early vocal learning reduces song preference and imitation in zebra finches**

Natalie R. Pilgeram, Nicole M. Baran, Aditya Bhise, Matthew T. Davis, Erik N. K. Iverson, Emily Kim, Sumin Lee, Carlos A. Rodriguez-Saltos, and Donna L. Maney

## SUPPLEMENTARY TABLES S1-S3 AND FIGURE S1

**Supplementary Table S1. Effects of oxytocin antagonist (OTA) on pupils' behaviors during tutoring sessions.** Behaviors were scored either as point counts per min or as a percentage of the trial, as indicated. The "tutor zone" was the area of the pupil's cage closest to the tutor, defined as within 12 cm of the wall facing the tutor's cage. "Pecks to tutor" is the number of times the pupil pecked at that wall.

| | | Control | | OTA | | Effect of OTA | | |
|---|---|---|---|---|---|---|---|---|
| | **Behavior** | **M** | **SD** | **M** | **SD** | $X^2$ | ***p*** | $d_{av}$† |
| *Approach* | **Time in tutor zone** (percent of trial) | 69.4 | 28.7 | 79.9 | 27.3 | 1.68 | 0.195 | 0.38 |
| | **Pecks to tutor**/min | 0.10 | 0.12 | 0.03 | 0.05 | 5.02 | **0.025*** | **0.83*** |
| *Attention* | **Flying bouts** (two-footed wall contacts/min) | 0.22 | 0.40 | 0.26 | 0.47 | 0.02 | 0.878 | 0.11 |
| | **Vocalizations**/min | 0.51 | 0.76 | 1.22 | 3.06 | 0.71 | 0.400 | 0.37 |
| | **Preening** (percent of trial) | 2.96 | 2.54 | 1.61 | 1.51 | 9.64 | **0.002*** | 0.67 |

†Effect size is calculated as Cohen's $d_{av}$, which takes into account the within-subjects design (see Methods)

**Supplementary Table S2. Effects of oxytocin antagonist (OTA) on tutor song rate**. The effect of treating the pupil with OTA on the song rate of the tutor (songs per minute of trial) was tested using two models. In the first (controlling for pupil ID), the song rates of the control and OTA tutors were compared within-pupil. In the second (controlling for tutor ID), the song rates were compared within-tutor, between the sessions with a control-treated pupil and those with an OTA-treated pupil.

| | Control | | OTA | | Effect of OTA | | |
|---|---|---|---|---|---|---|---|
| **Model** | **M** | **SD** | **M** | **SD** | $X^2$ | ***p*** | $d_{av}$† |
| *Controlling for pupil ID (n=9 pupils)* | 1.04 | 1.37 | 0.55 | 0.72 | 4.84 | **0.028*** | 0.47 |
| *Controlling for tutor ID (n=8 tutors)* †† | 1.17 | 1.41 | 0.52 | 0.77 | 7.03 | **0.008*** | 0.59 |

†Effect size is calculated as Cohen's $d_{av}$, which takes into account the within-subjects design (see Methods). *$p < 0.05$ or $d_{av} > 0.8$ (large). ††The sample size is 8 tutors for the second model because the 9$^{th}$ pupil had two unique tutors that tutored in only one condition each.

**Supplementary Table S3. Effects of oxytocin antagonist (OTA) on song learning.** The effect of treating the pupil with OTA on the quality of imitation of tutor song was tested using two measures of song similarity. In the first analysis, we used the maximum similarity score, out of 3-5 exemplars of song from each pupil. In the second, we used the average similarity score, over the same exemplars, for each pupil.

| | Control | | OTA | | Effect of OTA | | |
|---|---|---|---|---|---|---|---|
| **Measure of song similarity** | **M** | **SD** | **M** | **SD** | $X^2$ | ***p*** | $d_{av}$† |
| *Maximum similarity* | 0.84 | 0.12 | 0.63 | 0.24 | 3.66 | 0.056 | **1.15*** |
| *Average similarity* | 0.74 | 0.10 | 0.57 | 0.23 | 3.06 | 0.080 | **1.05*** |

†Effect size is calculated as Cohen's $d_{av}$, which takes into account the within-subjects design (see Methods). *$p < 0.05$ or $d_{av} > 0.8$ (large).

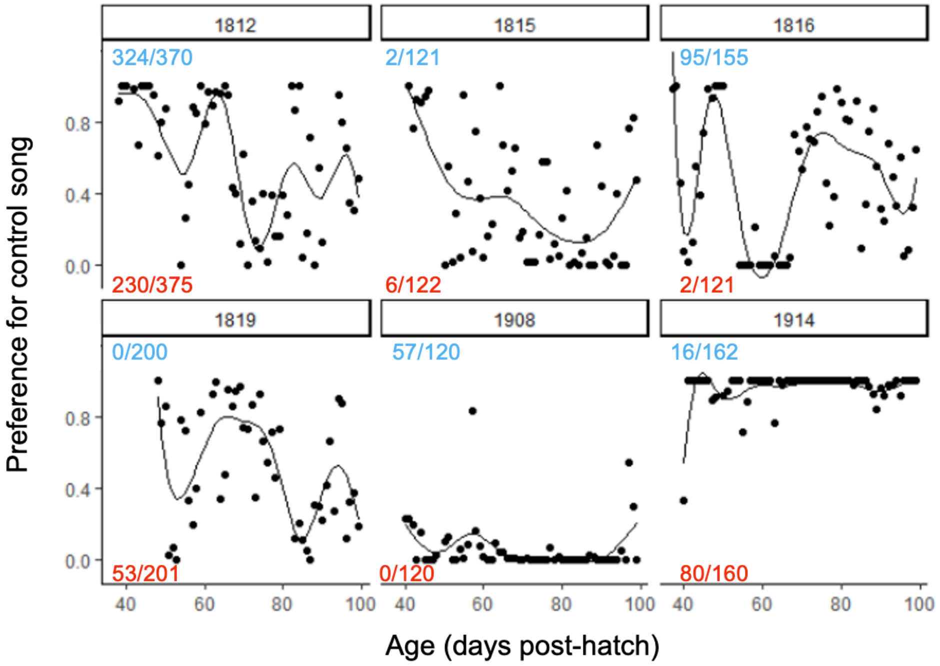


**Supplementary Figure S1. Individual trajectories of song preference.** The developmental trajectories of preference are shown for the six pupils in this study that completed the key-pressing assay. The preference for control song was calculated as the proportion of presses for the key associated with that song. The dots in each plot are the daily preference scores for each bird. A smooth trajectory was calculated by applying LOESS to the datapoints. Variation in the shapes of the trajectories is typical of developmental processes [1]. Despite the original data consisting of proportions, and thus bound to the interval (0-1), LOESS may fit values slightly outside that interval. The numbers of live songs heard, relative to the total number of songs heard are shown in blue at the top of each plot for the control tutor and in red at the bottom of each plot for the OTA tutor.

1. Ramsay, J. O. & Silverman, B. W. *Functional Data Analysis* (Springer, 1997).
